# Clinical significance and main parameters promoting the breast‑feeding strategy (Review)

**DOI:** 10.3892/mi.2024.138

**Published:** 2024-02-09

**Authors:** Eleni Nixarlidou, Chrysoula Margioula-Siarkou, Aristarchos Almperis, Eleftherios Vavoulidis, Antonio Simone Laganà, Konstantinos Dinas, Stamatios Petousis

**Affiliations:** 1 2nd Department of Obstetrics and Gynecology, Aristotle University of Thessaloniki, 54624 Thessaloniki, Greece; 2 Unit of Gynecologic Oncology, ARNAS ‘Civico-Di Cristina-Benfratelli’, Department of Health Promotion, Mother and Child Care, Internal Medicine and Medical Specialties (PROMISE), University of Palermo, I-90127 Palermo, Italy

**Keywords:** breast milk, breastfeeding, exclusive breastfeeding, breastfeeding establishment

## Abstract

Breastfeeding provides numerous nutritional and immunological benefits, promotes neurological and cognitive development, and protects against chronic and infectious diseases, rendering it beneficial to the survival and well-being of infants. According to international recommendations, infants should be exclusively breastfed for the first 6 months. However, despite global health recommendations and funding initiatives, exclusive breastfeeding rates remain low worldwide. A number of studies attribute the low rates to factors that can be grouped into demographic, psychosocial, economic and midwifery factors, and outline the profile of each mother who opts to exclusively breastfeed her infant. In addition, the number of previous pregnancies, induced labor, the use of epidurals at birth or the possibility of the newborn being delivered prematurely, and the need for admission to an intensive care unit are the factors that reduce the likelihood of exclusive breastfeeding. Further research is required to understand the factors influencing the initiation and maintenance of exclusive breastfeeding, as international interventions have been ineffective. The aim of the present review was to provide an up-to-date summary of these various factors in an aim to assist health care professionals and policy makers in developing effective interventions with which to promote and support exclusive breastfeeding.

## 1. Introduction

Nutritional needs and recommendations for optimal intake have been a matter of debate with the main focus on the preferred length of breastfeeding duration and associated benefits. The health benefits of breastfeeding for both the mother and baby are widely acknowledged. Although the rate of exclusive breastfeeding is increasing worldwide, it continues to remain at low levels (1). Various organizations, such as the United Nations Children's Fund (UNISEF), the World Health Organization (WHO) and the American Academy of Pediatrics (AAP) recommend that ideally, all infants should be exclusively breastfed for the first 6 months after birth (2). Exclusive breastfeeding indicates that the infant receives only breast milk and no other form of complementary formula food, including drinking water, with the exception of vitamins, minerals or medication (3). The WHO defines breastfeeding as the optimal nutritional intake for newborns and restricts it to the first hour after birth. Since 2001, exclusive breastfeeding has been recommended as the gold standard in infant nutrition for the first 6 months of life, followed by continued breastfeeding for 2 years or more, if desired by both the mother and her newborn, with complementary feeding from the first 6 months of life (4).

Compliance with all WHO recommendations is an important reminder that will help reduce neonatal morbidity and actively contribute to child survival, as human breast milk (HBM) acts as a safety net through various mechanisms. It is well known that that due to the sharing of antibodies, infants are protected from short- and long-term diseases, while breastfed babies have a lower risk of developing asthma, obesity, type 1 diabetes and sudden infant death syndrome (5). The benefits of breastfeeding are numerous both for the mother and child. However, the targets for exclusive breastfeeding have yet to be met.

The aim of the present narrative review was to document the epidemiological and obstetric factors associated with the establishment of this type of diet following delivery, and its maintenance by the mother and infant following hospital discharge.

## 2. The unique composition of breast milk

HBM is a unique infant nutritional regimen due to its various components that provide essential nutritional support for early human growth and development (6). Adapted to the needs of the infant, it is a dynamic fluid containing numerous immunological and anti-inflammatory substances, the composition of which changes over the course of lactation to fulfil the infant's nutritional needs (7). This bioactive potential of HBM is the exclusive prerogative of breast milk and is not present in any commercial formula.

HBM mainly contains basic nutrients, such as proteins, lipids, carbohydrates, vitamins, minerals and bioactive molecules, with the exception of water, which accounts for 87-88% (8). These components include immune factors, such as antibodies, immunoglobulins, antimicrobial peptides, growth factors, lysozyme, white blood cells, microRNAs and human milk oligosaccharides, which play a pivotal role in the development of the infant's immune system and provide an adequate immune barrier against pathogens through the development of its microbiome (8,9).

Finally, certain components of breast milk, such as microRNAs affect gene expression at the post-transcriptional level in newborns, and thus affect their health status at numerous levels (10).

## 3. Benefits of exclusive breastfeeding for mothers and babies

Extensive research has been conducted over the years on the benefits that exclusive breast feeding brings to both mothers and babies. Key benefits include the components of breast milk and the early mother-infant contact that occurs within the first hour of life, which is an essential measure to reduce neonatal morbidity and improve child survival (11). Commencing breastfeeding within the first hour after birth provides the best possible start in life and increases the chances that newborns will receive colostrum, the first milk produced by the mother. Colostrum is widely known for its immunological, anti-inflammatory and anti-infective effects, as well as its regulatory role in early intestinal colonization and immune development. It contains vitamin A and potent levels of antibodies, such as epidermal growth factor, which accelerates the development of intestinal mucus, as well as a variety of immunological bioactive factors that prevent pathogens from invading the intestinal mucosa of newborns (12). In healthy infants, the gut microbiome undergoes significant changes over time until around the age of three, at which point it stabilizes and resembles the microbial composition found in adults, characterized by anaerobic bacteria. Disruptions in this microbial environment are linked to increased vulnerability to autoimmune disorders such as diabetes, inflammatory bowel disease, allergies (atopy) and various other health conditions. The prevailing consensus acknowledges that the HBM microbiome plays a role in the colonization of the infant's gut. Breastfeeding facilitates the transfer of the HBM microbiome into the infant's gut, acting as a form of inoculation. Research indicates that certain strains of species, including *Lactobacillus* and *Bifidobacterium*, are present in both the HBM of the mother and the feces of the infant. This finding supports the notion that HBM contributes to the vertical transmission of beneficial bacteria (13-15). HBM supports the psychomotor, neurological cognitive and sensory development of infants. Consistent evidence suggests an advantage in cognitive outcomes in infants who have ever been breastfed or breastfed for longer periods of time compared to infants who have never or who have been breastfed for a short period (16). Furthermore, according to the results of a recently published review, providing human milk, particularly to preterm infants, while leading to a slower weight gain compared to formula feeding, is linked to an improved restoration of body composition. This improvement is achieved by fostering the deposition of fat-free mass, potentially resulting in enhanced metabolic and neurodevelopmental outcomes (17). Another literature review published in 2023 revealed that the intake of HBM macronutrients and bioactive components was implicated in the development of the infant and tend to have appropriate growth patterns, achieving healthy weight gain and growth milestones aligned with their individual growth curve (18). Furthermore, compelling evidence suggests that HBM is markedly associated with the risk of developing diarrhea and respiratory infections, including hospitalizations for these diseases (6,19). Infants who are breastfed in the long-term have also been shown to have lower rates of morbidity, mortality, dental dysfunction and higher rates of obesity than infants who were breastfed for shorter periods of time or not at all (20).

Breastfeeding is considered to be equally critical for mothers, providing a number of advantages, as it accelerates the return to pregestational fitness levels and markedly contributes to weight reduction during pregnancy (21). Previous research carried out on 314 Mexican mothers demonstrated that those who exclusively breastfed for a minimum of 3 months experienced a decrease in weight by 4.1 kg in contrast to those who chose not to breastfeed. This feature helps lactating women get back their previous image which, in turn, decreases the likelihood of negative emotional influences that could impede both lactation and the consistent practice of breastfeeding (22). It also reduces the risk of developing cardiovascular diseases, such as high blood pressure, type II diabetes and metabolic disorders. This fact is due to metabolically more active visceral or intra-abdominal fat tissue, which can be mobilized during the lactation period and continues to occur during breast-feeding. Several studies have pointed out the beneficial role of breastfeeding against the risk of developing breast and ovarian cancer. The reduction in estrogen levels during the lactation period decreases the rate of cell proliferation and differentiation and consequently reduces the probability of cells-mutations arising in mammary tissues (23,24).

Osteoporosis is another key aspect in which breastfeeding contributes with a beneficial and protective role, since lactating women tend have a bone mass with a higher mineral density. An explanation for this is the compensatory mechanisms that increase the calcium concentration in the blood and thus compensate for the loss during breastfeeding (21). Breastfeeding also plays a pivotal role in woman's mental health by reducing the incidence of postpartum depression. It is well known that a great number of all women can develop signs and symptoms of depression within 12 weeks postpartum due to the regulation of hormone secretion. These women have been found to have lower levels of oxytocin compared to others, which turns out to be a crucial element in infant-maternal bonding (21,23,25). It has also been shown that HBM has a positive impact not only on children's cognitive, but also on their social development, decreasing the proportion of social disorders, such as attention deficit hyperactivity disorder, resulting in more social and extroverted behavior (6,26).

## 4. Breastfeeding rates worldwide

Although exclusive breastfeeding is the most nutritious method of feeding infants and offers numerous benefits for both the mother and child, breastfeeding rates are remain low worldwide. The WHO has set an achievable minimum target of 50% of infants worldwide being exclusively breastfed. A goal that was never reached, as Europe had one of the lowest rates of breastfed infants, at 25% from 2006 to 2012 (6,27). In the USA in 2017, a rate of 25.6% of women who exclusively breastfed their infants for up to 6 months was recorded (28). Finally, in contrast to Europe and the USA, South Asia and Africa performed better in breastfeeding their newborns in 2016(6) due to the low- and middle-income status of many of their countries, such as India, Bangladesh, Ethiopia and Sudan, with rates ranging between 43 and 51% (29).

## 5. Barriers to breastfeeding

Efforts to promote breastfeeding have been ineffective, despite international interventions by large organizations. Global studies appear to link low rates of breastfeeding in newborns to factors influencing the initiation of exclusive breastfeeding (29,30). These factors can be grouped into demographic, psychosocial, economic and midwifery factors, and describe the profile of every mother who chooses to exclusively breastfeed her infant. The main factors are as follows:

### Maternal age

According to the literature, there is a positive association between an increased maternal age and breastfeeding duration (26). Older mothers appear to be more aware of the beneficial effects of breastfeeding on themselves and their infants. Of note however, research in the general population has demonstrated that maternal age does not appear to have a significant effect on breastfeeding duration (30).

### Maternal education level

There are numerous studies demonstrating that mothers with a high level of education exclusively breastfeed more often than mothers without proof of education They have also been found to breastfeed for longer periods of time compared to mothers with a lower level of education (31). Research has also shown a positive association between a high level of education of the husband and the average duration of breastfeeding (32), while there is some conflicting research indicating that high levels of parental education do not appear to have a beneficial effect or to be associated with a longer duration of breastfeeding (33).

### Socio-economic status and other demographics of the mother and her spouse play a vital role in breastfeeding practice

A satisfactory economic income, a higher social class, marital status (married), spouse support, extended family and the social environment appear to have a positive influence on the initiation and duration of breastfeeding, according to previous studies (34,35). The religion and ethnicity of the mother (35), regardless of their country of residence, also appear to be part of the equation, while other researchers have demonstrated that all these factors and characteristics do not appear to have a significant impact (33).

### Maternal employment and maternity leave

The total number of hours a working mother is employed has been shown to have a significant effect on breastfeeding duration, as part-time employment is associated with a longer breastfeeding duration (36). Returning to work and the length of post-natal and maternity leave in general, are another common barrier that appear to determine the duration of breastfeeding. Each country has its own regulations for maternity and paternity leave, the period after childbirth when the mother has to return to work. A leave of >6 months following childbirth has been found to be associated with a significantly higher rate of exclusive breastfeeding (37).

### Smoking

Smoking during pregnancy and breastfeeding is considered a critical barrier in initiating and maintaining breastfeeding compared to demographically similar non-smokers (38). This negative association is due to the conscious decision of the smoking mother not to breastfeed, being aware of the risks this harmful habit poses to her child.

### Maternal intention to breastfeed

A mother's intention to breastfeed can be influenced by a number of factors, including age, race, educational level, family economic status, awareness of the beneficial effects that breastfeeding has on the infant, previous breastfeeding experience, working hours, maternity leave and family support. Additional difficulties may arise postpartum that may negatively affect the mother's willingness to breastfeed (39), such as low self-esteem, doubtfulness in her ability to breastfeed, incorrect breastfeeding technique, inadequate information and support from midwives, uncertainty about the quantity and quality of milk, inadequate infant weight gain and embarrassment when breastfeeding in public places, factors that can influence a mother's intention to opt to exclusively breastfeeding her newborn (39,40). The preferred and ultimate duration of breastfeeding can be prolonged by the early identification of the parameters that negatively affect a mother's intention to breastfeed, by encouraging her to continue breastfeeding despite difficulties (40). In all these areas, the family and friendly environment can be of great assistance by helping the mother and counseling her to overcome obstacles, while in some cases, the support of expert medical and nursing staff can have substantial contribution (40,41).

### Maternal prior breastfeeding experience

According to the literature, mothers who have breastfed their first baby in the past are more likely to breastfeed their other infants as well for a longer period of time than mothers who are breastfeeding for the first time (31). It appears that the experience of breastfeeding the previous baby helps the mother to overcome all the difficulties she encounters in the maternity hospital and after discharge. Moreover, compared to mothers who did not breastfeed their previous baby, first-time mothers are more likely to initiate the practice at this point (40). This behavior may be attributed to the reasons that led mothers not to breastfeed their first child (e.g., smoking, rapid return to work, previous ‘traumatic’ experiences) persisting in the following pregnancies. The feeling of uncertainty regarding their ability to breastfeed or the adequacy and suitability of breast milk, make the decision to try again more difficult. In addition to this, once they begin breastfeeding, they give up more easily, which clearly translates into shorter breastfeeding durations.

### Types of birth

According to various studies, a cesarean section (C-section) has been proposed to be a strong predictor of the early initiation and establishment of breastfeeding (42). A C-section is a major barrier to the early attachment of the newborn to the mother's breast and therefore has a negative impact on the mother's decision to breastfeed her baby. Moreover, the planned C-section is considered the most critical factor affecting breastfeeding. It has been shown that women who had a vaginal birth were 3-fold more likely to commence breastfeeding early than women who had a C-section (43). Similar studies have shown that a C-section is also associated with a delayed initiation of breastfeeding compared to women who have given birth by normal vaginal delivery (42,44). These data may arise from the feelings of wound pain, stress and fear, particularly in emergency C-sections, delayed skin-to-skin contact between the mother and baby following birth, particularly in those with complications, and fatigue that women experience during the procedure, while the prolonged post-operative recovery time renders the decision even more difficult.

### Other factors

Other factors influencing breastfeeding include the following: i) Epidural anesthesia, used during normal childbirth to relieve pain and providing a numbing sensation during a C-section. Epidural drugs cross the placenta and may affect the infant's ability to breastfeed, as do obstetric drugs used during labor (45); ii) severe neonatal health issues following a C-section and epidural anesthesia require admission to a neonatal intensive care unit where the infant may be sedated and intubated, rendering breastfeeding impossible; iii) multiple pregnancies are more likely to require neonatal intensive care. This is due to their low birth weight and respiratory issues; iv) prematurity with a gestational age of ≤34 weeks and the inability of the infant to be breastfed due to the insufficient development of the suckling reflex; v) anatomical facial anomalies (e.g., Pierre Robin syndrome) that cause problems with milk swallowing and require special handling of the infant during feeding; vi) the marketing and promotion of breast milk substitutes. Breastmilk replacements or formula milk as they are commonly known are commercially available milk replacements that are heavily promoted through aggressive advertising campaigns to be a healthier and more nutritious food for the baby, despite evidence of the contrary. The majority of parents strive to adopt formula milk as a panacea for various issues in their newborn, from colic to allergies (46). A summary of the factors favoring or hindering breastfeeding is provided in Table I.

## 6. Conclusions and future perspectives

Breastfeeding, more than any other preventive measure, has the greatest potential effect on child health and mortality (46). Parameters that promote or inhibit the initiation of breastfeeding have been identified in numerous studies worldwide (46). Thus, some important variables favoring the maintenance of exclusive breastfeeding during hospitalization and following discharge are early onset, partner and social support during pregnancy, delivery and postpartum, breastfeeding of previous children, women's educational attainment, economic solidity and part-time employment of the breastfeeding mother. Conversely, obstetric factors associated with multiple pregnancies, induced labor, the use of epidural anesthesia at birth, or possible preterm delivery of the newborn and the need for admission to an intensive care unit significantly reduce the likelihood of exclusive breastfeeding.

The difference in maintaining exclusive breastfeeding is reflected in the support and advocacy strategies recommended by the WHO, UNICEF and other partners. It is a global advocacy initiative working to increase political commitment and investment in breastfeeding as a cornerstone of child nutrition, health, and development. One of the most crucial strategies in this direction is the establishment of the International Code of Marketing of Breast-milk Substitutes (47). Its main objective is to ensure the safe and adequate feeding of infants and to protect and promote breastfeeding, as well as to ensure the appropriate use of breastmilk substitutes, based on adequate information, when necessary (47).

The introduction and implementation of maternity leave makes it easier for working mothers to focus on breastfeeding their children through flexible working arrangements (48). These policies should be based on the principles of equality, human rights and public health and enabled by a system-wide political and social commitment to breastfeeding.

The implementation of practices in healthcare systems that promote exclusive breastfeeding, e.g., early skin-to-skin breastfeeding, kangaroo care, shared breastfeeding, exclusive breastfeeding during the entire clinical stay of the mother and finally cup-feeding, are interventions that pave the way in this direction (48,49).

It is clear that exclusive breastfeeding can be a focus for multilevel and multifaceted interventions, and that at the community level, the basic education of healthcare providers could cover the entire antenatal and postnatal period of new mothers, supporting and defending their right to exclusively breastfeed their newborns (45).

## Figures and Tables

**Table I:** Summary of factors affecting breastfeeding.

Factors favoring breastfeeding	Factors hindering breastfeeding
Increased maternal age	Smoking
Higher maternal education level	Cesarean section
Higher socio-economic status of couple	Epidural anesthesia
Longer maternity leave	Maternal employment
Maternal intention to breastfeed	Severe neonatal health problems
Maternal prior breastfeeding experience	Multiple pregnancies
	Prematurity
	Anatomical facial anomalies (e.g., Pierre Robin syndrome)
	Marketing and promotion of breast milk substitutes

## Data Availability

Not applicable.
